# Evaluation of Newly Introduced Bioactive Materials in Terms of Cavity Floor Adaptation: OCT Study

**DOI:** 10.3390/ma14247668

**Published:** 2021-12-12

**Authors:** Heba B. Abdel-Maksoud, Aziza W. Bahanan, Lujain J. Alkhattabi, Turki A. Bakhsh

**Affiliations:** 1 Restorative Dentistry Department, King Abdulaziz University, P.O. Box 80209, Jeddah 21589, Saudi Arabia; taabakhsh@kau.edu.sa; 2 Restorative Dentistry Department, Faculty of Dentistry, Suez Canal University, Ismailia 41611, Egypt; 3 Faculty of Dentistry, King Abdulaziz University, P.O. Box 80209, Jeddah 21589, Saudi Arabia; bahananaziza@gmail.com (A.W.B.); lujain.alkhattabi109@gmail.com (L.J.A.)

**Keywords:** adaptation, bioactive, composite, gap, OCT

## Abstract

Objective. The aim of the present study was to evaluate the adaptation of newly introduced bioactive restorative materials to the cavity floor using cross-polarization optical coherence tomography (CP-OCT). Materials and Methods. Round class V cavities were prepared on the proximal surfaces of sixty non-carious human anterior teeth (0.5 mm depth × 4 mm diameter), which were divided into groups according to the restorative material (n = 15). In the VF group, Vertise flow composite (Kerr, Orange, CA, USA) was used, in the BF group, Beautifil II composite (Shofu, Koyoto, Japan) was used, and in the AB group, ACTIVA BioACTIVE composite (Pulpdent, Watertown, NY, USA) was used. Cavities were restored using the bulk filling technique and cured according to the manufacturers’ instructions. Then, the specimens were immersed in a contrasting agent, and image acquisitions were taken by CP-OCT to calculate the adaptation percentage by using an image analysis software. Results. B-scans showed a diffuse bright band of white pixels at the tooth-resin interface that was interpreted as a micro-gap present between the cavity floor and restorative material. The Kruskal-Wallis test showed a statistically significant difference between all tested groups with the AB group representing the least gap formation, followed by the BF group, and then the VF group, which demonstrated the highest gap formation. Conclusions. In class V cavities, better adaptation to the cavity floor can be obtained when using ACTIVA BioACTIVE more than Vertise flow and Beautifil II composites. In addition, CP-OCT is considered a non-destructive imaging tool that helps in evaluating the quality of the tooth-restoration interface when bioactive composites are used.

## 1. Introduction

New technologies were directed to reduce microleakage, thus making it a very important and crucial subject for researchers and clinicians [1]. Manufacturers nowadays are in a continuous challenge trying to provide the dental market with both dental adhesives and restorative materials that result in a gap-free as well as bacteria-free tooth/restoration interface [2].

Bioactive restorative materials were introduced to the dental market with the polymerization shrinkage problem still existing unfortunately as the main reason for marginal gap formation and subsequent marginal leakage, both of which are not easily diagnosed neither clinically nor radiographically [3,4].

Optical coherence tomography (OCT) is known as a non-invasive and non-hazardous high-resolution imaging system that can give us tomographic and volumetric images of biological structures and non-metallic biomaterials at micron scale [5]. This new imaging diagnostic tool has demonstrated its ability in the field of restorative dentistry in identifying any defects or changes in restorations, enamel, and superficial dentin layer. It has been proposed that the variations in the backscatter strength signal from various dental systems are induced by the variability of tissue texture and orientation [6]. It is also considered a reliable method for diagnosing oral disorders. It was developed based on the concept of low-coherence interferometry; a laser source is projected over a sample, and the intensity of the backscattered signal from within the scattering medium is coupled with the reflected reference light from a mirror. Fringes of interference show depth-resolved details regarding the sample’s scattering and reflection of light. The signal from serial linear scans can be transformed into an image by software. OCT technology has made significant progress in recent years through the development of techniques of spectral discrimination, which provides a substantial increase in sensitivity over the traditional OCT time domain.

Cross-polarization optical coherence tomography (CP-OCT) is a functional modification of SS-OCT that is one of the latest applications of OCT imaging technology, which provides cross-sectional images with a higher resolution and speed compared to the conventional time-domain systems. This permits instant imaging of the dental hard tissue. It detects backscattered signals that are perpendicular to linearly polarized signals and reduce the specular reflections, which would enhance the visualization of dental biofilms, enamel demineralization, and remineralization.

Different dental materials used in restoring different classes of cavities were evaluated for cavity floor adaptation using CP-OCT in several studies. Others were concerned about detecting marginal defects of class II composite resin restorations based on radiographs. Moreover, some investigators compared microleakage as well as gap formation using four adhesive restorative procedures in class I cavities [3].

However, to the best of our knowledge, limited or even no studies have evaluated the cavity floor adaptation of these chosen bioactive materials using CP-OCT. Therefore, this in vitro study aimed to compare the newly introduced bioactive restorative materials used in restoring class V cavities in terms of cavity floor adaptation using CP-OCT. The null hypothesis in this study is that there is no difference in adaptation among the tested materials at the cavity floor.

## 2. Material and Methods

### 2.1. Materials Used

In this study, three types of recently introduced material were used; Vertise Flow composite (VF; Kerr, Orange, CA, USA; Lot Number: 7063158), Beautifil II (BF; Shofu, Kyoto, Japan; Lot Number: 051951) and ACTIVA BioACTIVE-RESTORATIVE (AB; PULPDENT, Watertown, NY, USA; Lot Number: 190513), and A universal dental adhesive (Tetric N-Bond Universal; Ivoclar Vivadent, NewYork, NY, USA; Lot Number: W91986).

The chemical compositions of the tested materials are listed in Table 1.

### 2.2. Sample Preparation

This work took approval from the Research Ethics Committee at King Abdulaziz University (184-11-19), according to the guiding principles for investigational methods found in the Declaration of Helsinki of the World Medical Association. After a pilot study, the sample size was calculated using a 0.05 alpha value and 80% power to detect a difference of 25% (PiFace, http://homepage.stat.uiowa.edu/~rlenth/Power/ (accessed on 22 November 2019). The common standard deviation within a group was assumed to be 18%. The estimated sample size for every group of all tested groups should be at least 9 [7]. Finally, sixty sound anterior teeth were selected and kept in distilled water until the testing time. Teeth were washed and cleaned from any tissue debris. Round class V cavities were prepared on the proximal surface (0.5 mm depth × 4 mm diameter). Each tooth was placed individually in a separate container with a given mark. A simple randomized equal distribution was carried out to divide teeth equally into three experimental groups: VF, BF, and AB. Cavities were restored using the bulk filling technique and cured according to the manufacturers’ instructions. In the BF and AB groups, cavities were bonded with Tetric N-Bond Universal adhesive (self-etch mode), photoactivated for 10 s, and then restored with Beautifil II and ACTIVA BioACTIVE composites, respectively. However, in the VF group, cavities were restored with the Vertise flow composite directly without bonding. All materials were photoactivated using a halogen curing unit (Optilux501, Kerr, USA; 500 mW/cm^2^ intensity). For standardization issues, all cavities’ preparations and restorations’ applications were completed by only one operator. During the bonding procedure, the adhesive bottle was primarily shacked to be sure of mixing of all components altogether. A micro-brush was used to uniformly apply the adhesive over the internal walls of the cavity followed by air drying using a 3-way syringe at a 20 cm distance away. The light curing tip was kept at a 2 mm distance from the surface of the cavity. The light intensity was regularly checked using a radiometer throughout the preparation of all samples. Afterward, the composite was extruded from the composite tube and covered to prevent polymerization. Then, an increment of composite was used to fill the cavity (bulk filling) using a plastic instrument. The excess of composite was removed, and a small glass slide was placed over the top of restoration to ensure the uniform packing pressure. This step was done to assure the structural integrity of the composite material during curing. 

### 2.3. Contrasting Medium Preparation

**Figure 1:**
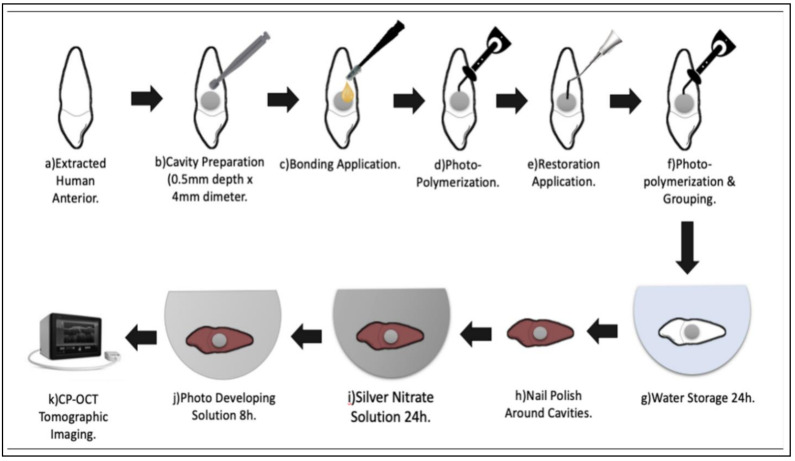
Schematic illustration of the specimen preparation, restoration, and CP-OCT imaging.

All specimens were kept at room temperature in hydrated condition for 24 h. The contrasting solution was formulated by dissolving 25 mg of silver nitrate in 25 mL of distilled water in a dark container. Therefore, a solution of ammonium hydroxide was inserted drop by drop to titrate the black solution until it turns translucent. Before immersion in the prepared solution for 24 h, all samples were coated by 1 mm around the margins with nail varnish, except for the restored region. Then, the specimens were washed with distilled water and placed under fluorescent light for 8 h in a photo-developing solution to turn the silver diamine ions into metallic grains [5]. A schematic drawing of the experiment is shown in Figure 1.

### 2.4. CP-OCT System

Prepared specimens were imaged using a CP-OCT system (IVS-300; Santec, Komaki, Japan). The technical specifications and imaging parameters are listed in Table 2. It employs a continuous wavelength scanning laser diode with fast scanning speeds (30 kHz) based near to 1310 nm, with a wavelength of around 100 nm. The system’s axial resolution and lateral resolution were respectively ≈12 and ≈30 μm. Within the literature, interferometric theories of the acquisition and processing of CP-OCT images are described [5]. The sample backscattered light was returned to the array, digitized in time scale, and then evaluated at each stage in the Fourier domain to show the depth-resolved reflectivity profile (A-scan), creating a raw data file (B-Scan). To build a two-dimensional cross-sectional picture, a gray-scale picture was processed from raw data on the B-scan. The B-scan picture resolution was 500 × 924 pixels equivalent to 8.18 mm (x, z) of 5 mm. Figure 1 provides a graphical description of the specimen planning, reconstruction, and OCT photography.

### 2.5. Tomographic Imaging and Image Analysis

Until tomographic analysis, all specimens were stored at room temperature for 24 h in a humid environment. The specimens were placed on a micrometer scale and perpendicular to the proximal surfaces were directed to the scanning probe and the laser beam. Many sequential B-scans were obtained along the restoration at a 250 µm interval distance. Gaps were detected as large signal intensities between the cavity and restoration in the form of bright white clusters of pixels. The lengths of cavity floor adaptation and micro-gap widths were calculated using a digital image software (ImageJ v. 1.45q; National Institutes of Health, Bethesda, MD, USA) [8].

The adaptation percentage parameter was defined as:

Adaptation% = [(total cavity floor length−sum of gap length)/total cavity floor length] × 100.

The imaging of specimens is shown in Figure 1.

### 2.6. Statistical Analysis

Statistical analysis was performed with IBM-SPSS Statistics Version 20 for Windows. Data were explored for normality using Kolmogorov-Smirnov and Shapiro-Wilk tests, data showed non-parametric (not-normal) distribution. The Kruskal-Wallis test was used to compare between more than two groups in non-related samples. Mann-Whitney was used to compare between two groups in non-related samples. The significance level was set at *p* ≤ 0.05 for both tests.

## 3. Results

**Figure 2:**
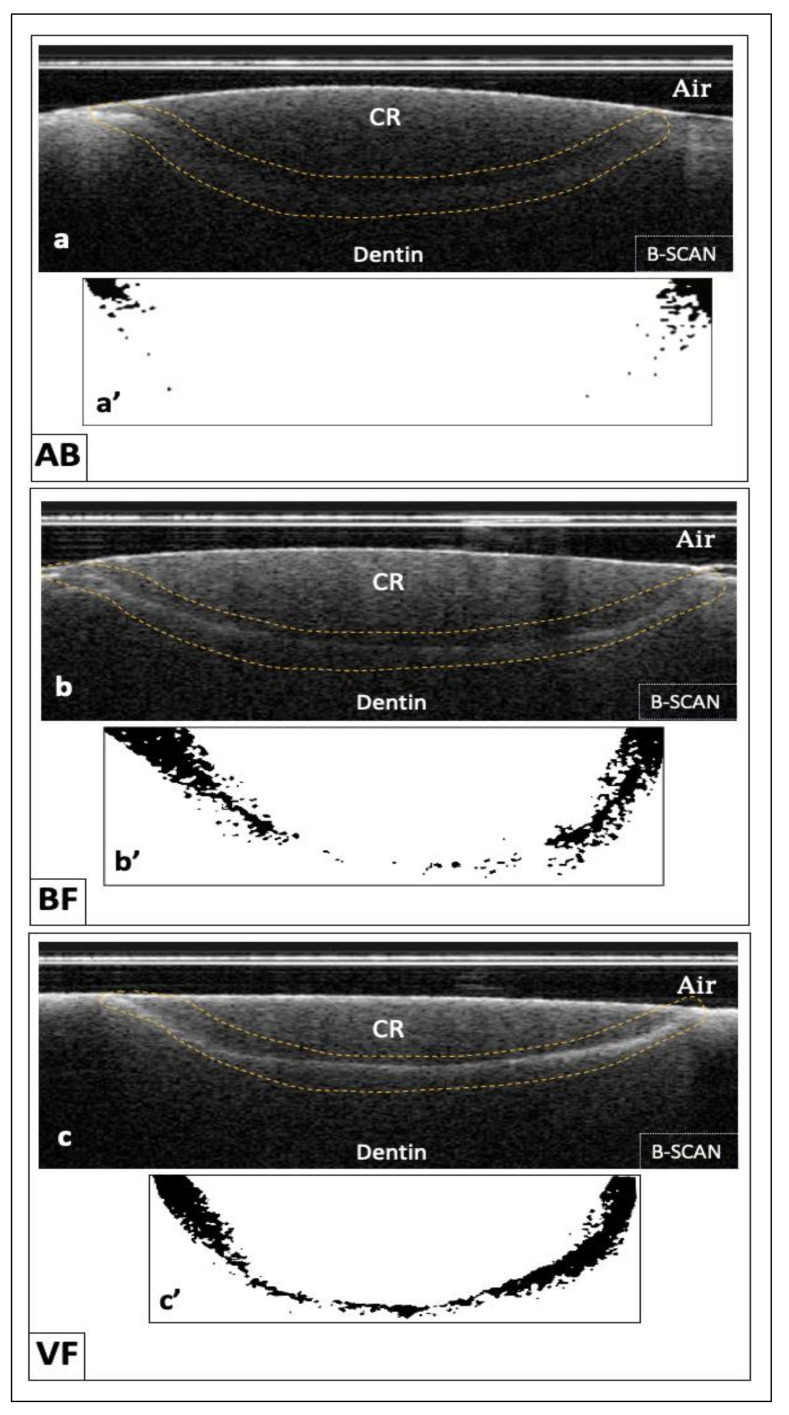
Representative B-scan images of the tested groups. (AB): In (**a**), the cavity floor in the AB group demonstrated slightly low signal intensity (dotted frame) without bright white cluster formation. After applying binarization function to the cavity floor (dotted frame in (**a**)), (**a’**) did not detect any changes in the background in the form of black pixels formation that represent gaps except for the cavity margins. (BF): In (**b**), the cavity floor in the BF group showed heavier signal intensity than the AB group (dotted frame) with some scattered bright white clusters formation. These clusters became scattered dark pixels on a white background in (**b’**). (VF): In (**c**), the cavity floor in the (VF) group showed the highest signal intensity among all the tested groups [dotted frame in (**c**)] with heavier clusters formation. After binarization, which facilitated gap quantification, (**c’**) black pixels were observed on the white background, and this was interpreted as gap formation.

Optical analysis of all cross-sections resulting from OCT reported higher signal intensity throughout the interface in some specimens. This was caused by the strong reflection of the diffusion of silver particles. These particles were detected as dark pixels in the binary image and represented the micro-gap between the cavity floor and the restorative material. On the other hand, some specimens reported very minimal brightness at this interface, which indicated the presence of a good interfacial seal. Representative B-scan images obtained by the CP-OCT of each group with binary images of the interface are shown in Figure 2. After analysis, the collected data showed a statistically significant difference in the gap percentage between the groups (*p* < 0.001). The highest gap percentage was found in the VF group (86.65% ± 18.67) followed by the BF group (84.41 ± 17.06), whereas the AB group showed the lowest gap percentage (58.18 ± 24.62). A statistically significant difference was found between the AB group and each of the VF and BF groups (*p* < 0.001). A statistically significant difference was found between the VF and BF group (*p* = 0.037)**.** The average gap percentage values for all groups with their standard deviations are presented in Table 3.

## 4. Discussion

In adhesive restorations, interfacial defects can be missed easily or only detected at later stages when a conservative treatment approach cannot be implemented [9]. Therefore, it is very important to properly evaluate the interfacial tooth/restoration interface for the occurrence of these defects. Class V cavities are to some extent easier than other preparations [10]; they represented an optimum evaluation for material adaptation to cavity floor in this work. They are also commonly shown among adults and elderly patients [11]. Clinical marginal quality assessments for intraoral restorations are routinely conducted in dental practice; however, the replacement of existing restorations and the decisions associated with treatment planning are very subjective [12]. Thus, providing the dental clinics with some tools such as OCT to quickly diagnose the tooth restoration interface is a dream that will dramatically change the future of evaluation of existing restorations. However, CP-OCT is still far away to be used in clinical practice.

OCT has been used extensively in caries treatment, remineralization control, vertical root fracture, and crack identification. It allowed quantitative analysis of the adaptive actions of micron-scale dental restorations in two-dimensional (2D) and three-dimensional tomograms without cutting or processing. Also, it can display material adaptation at the micron scale [13].

In this study, different types of restorative materials were used in restoring class V cavities. They were examined to evaluate cavity floor adaptation using CP-OCT. It investigates the adaptation of the restorative material to the cavity floor through precise spotting of all gaps between tooth and restoration surfaces all the way along through the interface.

After restoring the prepared cavities with the assigned restorative material, an ammoniacal silver nitrate solution was used to fill in all the spaces present between the tooth dentin and the surface of the material. This space is the gap that is targeted to detect. There are two different forms of reflection: specular and diffuse. These spaces or gaps shown in the B-scans in the present study were presented in the form of bright white clusters of pixels, which is different from specular one. The latter is based on the flatness of the surface of the image. It is barely seen with the CP-OCT, but it is prominently seen in the SS-OCT.

Bioactive composites were claimed by manufacturers to provide a perfect bacteria-free gap-free seal at the tooth/restoration interface. AB is a resin-modified glass ionomer cement (RMGIC) enhanced with ‘rubberized’ resin according to the manufacture. Some properties of this material such as its flexural strength were shown to be similar to that of flowable and bulk-filled resin composites. Moreover, wear was shown to be identical to that of a composite resin [14].

It is listed by the manufacturer as one of the first permanent dental restorations to incorporate bioactivity by responding to changes in the oral environment. It contains glass fragments and a layer of hydrophilic ionic resin that facilitates the absorption of multiple ions such as calcium, phosphate, and fluoride, which in turn leads to changes in the pH of the oral cavity. Consequently, at the tooth-material interface, which is our main concern, the restorative material will show adequate properties to provide a high-quality seal that is resistant to oral fluid contamination [15]. The exact composition of AB was not disclosed until the time of this study. Benetti et al. in 2019 confirmed the same fact, too. Therefore, we tried to find the good point in this material regardless of its difference in composition from BF. The combination between self-etched adhesive TN and bioactive AB results in a significantly superior adaptation in the present study. This comes along with Benetti et al. in 2019 [14]. They reported good marginal adaptation results with both enamel and dentin. Our results coordinate too with those of Omidi et al. [16] and Kaushik and Yadav [17]. Again, Hughes et al. [18,19] reported a good performance of AB.

AB and BF were bonded with TN self-etch. It is a single component water-based adhesive with both hydrophilic and hydrophobic monomers as well as a solvent. VF is a self-adhering composite that was used without bonding. A significant difference resulted between all the materials. AB showed a significant difference from each of the two other groups. It showed the least gap percentage that is interpreted into perfect adaptation to the cavity floor. In addition, a significant difference was found between BF and VF, with the latter showing the least cavity floor adaptation. Both AB and BF are bioactive composites, but they differ in terms of their filler content. Therefore, the null hypothesis was rejected.

Bonding had a great impact on the results of the present study. Both AB and BF restorations were bonded to the tooth structure; however, VF composite was not. The manufacturers claimed that VF is a self-adhering composite, which renders it time saving. This occurs through eliminating the bonding procedures’ steps. However, it showed the least adaptation to the floor of the cavity, resulting in the worst clinical behavior when compared to the other groups that were bonded to the tooth structure. This comes in accordance with Peterson et al. in 2018, who stated that self-adhesive composites resulted in a significantly lower shear bond strength to enamel and dentin than conventional flowable composites after aging [20].

In addition, the restoration of class V cavities with self-adhesive composites resulted in a significantly higher number of restorations’ loss or even unacceptable loss in another study, which emphasized the worse clinical behavior of these types of materials [21].

The materials’ composition is another factor that has a crucial influence on the restoration performance in the present study. This includes the matrix composition, namely the functional monomers as well as the filler content. The difference in composition between the materials used in the present study verifies the significant difference in their adaptation. Regarding VF, which is a self-adhesive composite, the interaction between these types of composites and enamel and dentin was recorded to significantly differ in many studies [22]. This composite has two opposing influencing criteria. Firstly, its matrix includes glycerol phosphate dimethacrylate (GPDM) monomers. This is a unique characteristic compared to other self-adhesive composites. Adding to this, the HEMA present in its matrix increases its wetting ability. On the contrary, these monomers have a much greater tendency to absorb water, which helps in matrix swelling and in turn the polymer chains destruction [23].

Secondly, its high filler content results in a relatively high viscosity, which in turn decreases its penetration ability. Memarpour et al. reported that VF achieved higher shear bond strength values to both enamel and dentin when bonded with an adhesive rather than when used alone [24]. They stated that using a bonding agent enhances the resin intermingling through increasing the micro porosities within dentin [25,26]. This confirms our results regarding the significantly higher gap percentage in the VF group (non-bonded group) when compared to the other tested groups (bonded groups).

However, an apparent relatively less potentiality to chemically bond with the dentin hydroxyapatite was reported in another study [27,28]. In addition, studies showed obvious thin sparse tags when evaluating the adhesive layer in the VF group in other studies. It was assumed that the intermingling between tooth tissue and adhesives is minimum in the presence of a smear layer [28]. Moreover, some studies reported a clinical failure of 66% of class V cavities restored with self-adhesive flowable composites.

The composite filler content was reported by several studies to greatly affect polymerization shrinkage [29,30]. In our study, AB group has a filler content of 56% by weight and showed superior cavity floor adaptation when compared to other tested groups; the BF group with filler content of 83.3% by weight and the VF group with 70% filler content by weight. Regarding the two bioactive composites groups, AB represented a significantly superior adaptation to the BF group. This comes in accordance with Bakhsh et al. in 2018, who reported the lower performance of resin composites with higher filler content. Volumetric shrinkage is influenced by the filler content in the material, too. The high filler content in VF could elevated the internal contraction stresses that might be responsible for the worst adaptation recorded in the present study.

Beautifil II was claimed by the manufacturer to have exceptional handling properties, an anti-plaque effect, surface hardness comparable to tooth enamel, and fluoride rechargeability. In the present study, it showed a significantly higher gap formation at the cavity floor/restoration interface. However, Kurokawa et al. in 2015 [19] stated that this material had excellent performance after three years of clinical service. They recommended its use in class I and II in selected patients. They explained their results by stating that using S-PRG fillers enhances fast fluoride-releasing by ligand exchanges between fluoride ions and counter cations, which are found in prereacted hydrogel. S-PRG fillers again cause a modulation that neutralizes pH after acidic exposure [31].

Under the limitations of the present study, the null hypotheses were rejected. Future studies should take place regarding the evaluation of the adaptation of the bioactive composite under several conditions that mimic the intraoral conditions such as thermocycling and pH cycling. In addition, the potential application of digital dentistry in evaluating restorative materials outcomes should be taken into consideration [32]. Moreover, since the role of occlusion is fundamental when restoring a tooth, dental practitioners should be aware of how to avoid potential disturbances [33] and, in addition to all mentioned techniques to test the behavior of these newly introduced materials, the role of stem cells in treating such materials must be pointed out [34].

## 5. Conclusions

Within the limitation of this in vitro study, OCT was a successful technique for detecting the internal adaptation of dental restorative material to the tooth structure. Since the manufacturers are trying to improve dental restorative materials, cavity floor adaptation has not been completely achieved. In this study, the result did not support the hypothesis that there is no difference in the adaptation among the tested materials in the cavity floor. It showed that the adaptation percentage results reported a statistically significant difference between the three restorative materials. Since Activa Bioactive composite restoration showed the least gap percentage, therefore, it is considered a good option for class V cavities. Additionally, long-term in vitro and in vivo studies are needed to properly evaluate the performance of bioactive composites. In addition, studies are to be conducted to manage recurrent caries using these materials.

## 6. Clinical Recommendations

Activa Bioactive is a promising restorative composite to be used by dental personnel, especially in cases where caries control is a must. Its good adaptation may help in cases where the control of oral hygiene is questionable.

It might be very beneficial to use an adhesive when restoring cavities with Vertise Flow self-adhering composite.

## Figures and Tables

**Table 1:** The chemical composition of the used materials in the study.

Material (CODE) Manufacturer	Composition *	Lot NO.
Vertise flow (VF) Kerr, Orange, CA	Matrix: GPDM, HEMA. Fillers: prepolymerized filler, nanosized ytterbium fluoride, 1 mm barium glass filler, 10–40 nm nanosized colloidal silica. Uncured methacrylate ester monomers. (70% by weight)	7063158
Beautifil II (BF) Shofu Inc., Koyoto, Japan	Bis-GMA, TEGDMA, S_PRG filler based on fluoroboroaluminosilicate glass, polymerization initiator, pigments, and others. Filler wight 83.3% Filler volume 68.6%	051951
ACTIVA BioACTIVE Restorative (AB) Pulpdent Corporation, USA	No bisphenol A, No bis-GMA, No BPA derivatives Filler by wight 56%	190513
Tetric N-Bond Universal (TN) Ivoclar/Vivadent	Bis-acrylamide derivative, Bis-GMA, amino acid acrylamide, hydroxyl alkyl methacrylamide diphenyl phosphine oxide, nano-fillers (SiO_2_), initiators, water, stabilizers	W91986

* Abbreviations: GPDM: glycerol phosphate dimethacrylate; HEMA: hydroxyethyl methacrylate; Bis-GMA: bisphenol glycidyl dimethacrylate; TEGDMA: triethylene-glycol dimethacrylate.

**Table 2:** Technical specification of CP-OCT system (cross-polarization OCT (CP-OCT; IVS-300, Santec, Japan).

Parameter	Specification
Wavelength	1330 ± 30 nm
Scan rate	30 ± 0.1 kHz
Axial resolution	≤12 μm (in air)
Lateral resolution (based on spot size)	30 ± 7 μm (in air)
System sensitivity	>95 dB
Lateral scan area	≥5 × 5 mm
Imaging depth	3 mm
Maximum output power	≥1 mW (near-infrared class 1 laser)

**Table 3:** Summary of the internal cavity floor gap percentage of the tested groups (mean ± standard deviation).

Groups	Gap (%)		St. Err
	Mean	SD	
VF	86.65% ^a^	18.67	0.04
AB	58.18% ^c^	24.62	0.05
BF	84.41% ^b^	17.06	0.03
*p*-value	<0.001 *		-

Means with different letters in the same column indicate statistically significance difference. * significant (*p* < 0.05), ns non-significant (*p* > 0.05).

## Data Availability

Data is available on request from the corresponding author.
